# Association of hepatitis C virus infection status and genotype with kidney disease risk: A population-based cross-sectional study

**DOI:** 10.1371/journal.pone.0271197

**Published:** 2022-07-08

**Authors:** Yi-Chia Chen, Hung-Wei Wang, Yun-Ting Huang, Ming-Yan Jiang

**Affiliations:** 1 Department of Internal Medicine, Chi Mei Medical Center, Tainan, Taiwan; 2 Renal Division, Department of Internal Medicine, Chi Mei Hospital Chiali, Tainan, Taiwan; 3 Renal Division, Department of Internal Medicine, Chi Mei Medical Center, Tainan, Taiwan; 4 Department of Pharmacy, Chia Nan University of Pharmacy & Science, Tainan, Taiwan; National Taiwan University Hospital, TAIWAN

## Abstract

**Background:**

Whether there is difference in kidney disease risk between chronic hepatitis C virus (HCV) infection and resolved HCV infection remains inconclusive. Additionally, the impact of different HCV genotypes on kidney disease risk is relatively unknown. Accordingly, we conducted a population-based cross-sectional study to investigate the association of HCV infection status and genotype on kidney disease risk.

**Methods:**

The study population were adult participants of 1999–2018 National Health and Nutrition Examination Survey in the United States. Chronic and resolved infection were defined as HCV seropositivity with and without detectable HCV RNA, respectively. HCV genotypes were classified into genotype 1, genotype 2, and other genotypes. Prevalent estimated glomerular filtration rate < 60 ml/min/1.73 m^2^ or urinary albumin creatinine ratio ≥ 30 mg/g was defined as kidney disease.

**Results:**

The average age of study population (n = 44,998) was 46.7±17.0 years with 49.8% being males. Compared with individuals without HCV infection (n = 44,157), those with resolved (n = 255) or chronic HCV infection (n = 586) had higher prevalence of kidney disease: 14.8%, 23.5%, and 20.1%, respectively (*p*<0.001). After adjusting for potential confounders, we found that both resolved (adjusted OR: 1.40, 95% CI: 1.02–1.93) and chronic HCV infection (adjusted OR: 1.26, 95% CI: 1.01–1.57) correlated to increased kidney disease risk compared with no HCV infection. Additionally, individuals with HCV genotype 1 (adjusted OR: 1.41, 95% CI: 1.09–1.82) but not genotype 2 or other genotypes had greater kidney disease risk compared with no HCV infection. Furthermore, we observed that genotype 1 had 2-fold higher kidney disease risk (adjusted OR: 2.20, 95% CI: 1.07–4.53) compared with non-genotype 1 HCV infection.

**Conclusion:**

Both resolved and chronic HCV infection, particularly genotype 1, were associated with higher kidney disease risk.

## Introduction

Hepatitis C virus (HCV) infection had been shown to associate with chronic kidney disease (CKD) [1]. When compared with HCV seronegative individuals, those with HCV seropositivity were more likely to have dipstick proteinuria and long-term risk of incident CKD [2,3]. Additionally, among United States (U.S.) veterans with baseline estimated glomerular filtration rate (eGFR) ≥ 60 mL/min/1.73 m^2^, those with anti-HCV antibodies or detectable HCV ribonucleic acid (RNA) were associated with higher risk of incident CKD and rapid decline in eGFR as opposed to seronegative individuals [4]. Furthermore, CKD patients with HCV seropositivity or HCV viremia had higher risk of kidney disease progression when compared with individuals without HCV infection [3,5,6].

Although the association between HCV infection and risk of kidney disease has been demonstrated in several studies, inconsistent results have also been observed. A claim data-based study showed that individuals with HCV seropositivity or viremia were not associated with increased risk of CKD compared with non-HCV infected individuals [7]. In addition, among the U.S. population, adults with anti-HCV antibodies were more likely to have albuminuria but not reduced GFR (< 60 ml/min/1.73 m^2^) [8]. Similarly, a meta-analysis also showed that HCV seropositivity was associated with greater risk of albuminuria but not with reduced GFR [9].

In previous studies, HCV infection was usually defined by either HCV seropositivity or presence of HCV RNA, of which HCV seropositivity may indicate resolved (without detectable HCV RNA) or chronic HCV infection (with detectable HCV RNA). However, several studies have demonstrated that sustained virological response from HCV treatment is associated with better kidney outcome [10,11], indicating that individuals without detectable HCV RNA have lower risk of kidney disease progression. Whether resolved and chronic HCV infections are similar in risk of kidney disease remain undetermined. Therefore, the use of HCV serostatus to define HCV infection in some of the previous studies may have led to information bias.

Although several HCV genotypes have been identified worldwide [12], studies on the relevance of HCV genotypes with kidney disease are relatively lacking and have produced conflicting results. While earlier studies showed no difference in kidney disease risk between different HCV genotypes [13,14], more recent studies have observed an association between different HCV genotypes and adverse kidney outcomes [15,16]. However, while one study showed that genotype 1 HCV infection was associated with greater risk of incident end-stage kidney disease (ESKD) but not genotype 2 [16], another study observed a stronger effect of genotype 2 than genotype 1 [15]. Whether there is difference in kidney outcome between different HCV genotypes remains inconclusive. Accordingly, we conducted a population-based study to investigate the association of HCV infection status and different HCV genotypes on kidney disease risk.

## Methods

Our data is obtained from National Health and Nutrition Examination Survey (NHANES) of the U.S., which is a series of health-related programs conducted by the National Center for Health Statistics [17]. The NHANES sample represents the noninstitutionalized civilian U.S. population residing in the 50 states and the District of Columbia. NHANES uses a complex, multistage, probability sampling design rather than simple random samples (https://wwwn.cdc.gov/nchs/nhanes/tutorials/module2.aspx). NHANES data are collected from survey participants using questionnaires on health-related topics in participants’ homes and a physical examination and laboratory tests in a mobile examination center, with data release in 2-year cycles. The data were publicly available on the website of National Center for Health Statistics. (Available from: https://www.cdc.gov/nchs/nhanes/index.htm.) and permission for publishing the analysis is not needed. Sample weights in NHANES have been constructed to adjust for non-response, oversampling, and non-coverage. Because of the thoroughness of its research methodology, NHANES data have been widely used over the years to reliably assess many diseases’ prevalence and risk factors. All NHANES protocols were approved by the research ethics review board of the National Center for Health Statistics, and all the participants provided written informed consent. The Research Ethics Committee of the Chi Mei Medical Center exempt review of this study.

**Fig 1:**
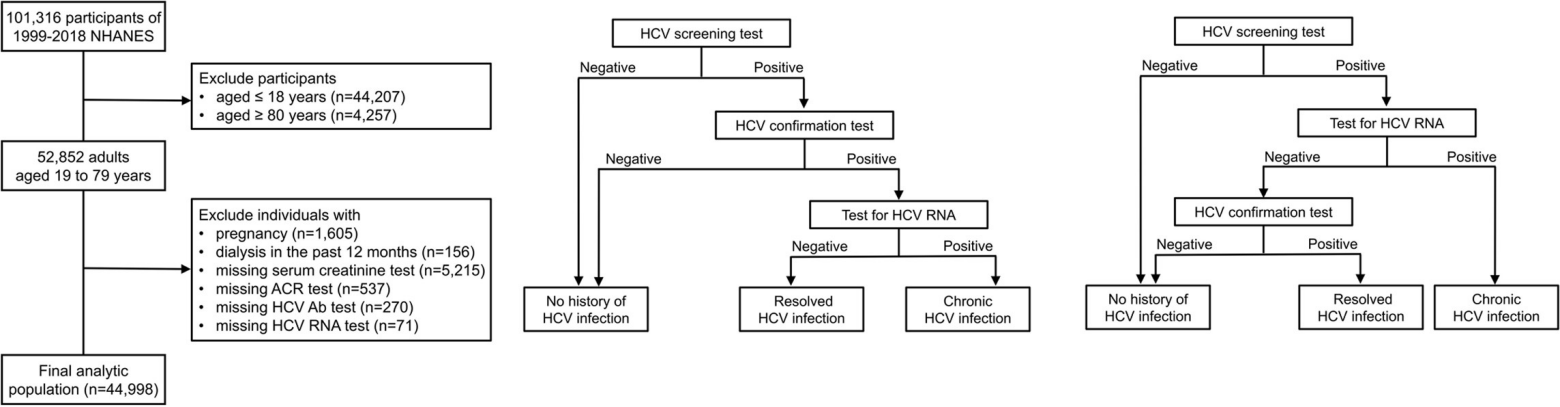
(A) Flow diagram of the study population selection. (B) Hepatitis C virus (HCV) testing algorithm in 2012 and earlier years. (C) HCV testing algorithm after 2013.

In this cross-sectional study, we merged the data from 10 discrete 2-year cycles (1999–2000 through 2017–2018) of the continuous NHANES to create our study population. We included adult participants aged 19 to 79 years (n = 52,852) and precluded individuals who were pregnant (n = 1,605), those who received dialysis in the past 12 months (n = 156), and those who did not have tests for serum creatinine (n = 5,215), urinary albumin and creatinine (n = 537), and HCV antibody (n = 270). Among individuals who were tested positive for anti-HCV antibody, we further excluded those who were not tested for HCV RNA (n = 71) because it is unable to differentiate resolved and chronic HCV infection. Therefore, the final analytic population consisted of 44,998 participants (Fig 1A).

The HCV infection status was defined by HCV antibody screening, confirmation, and/or HCV-RNA tests. Participants who were tested positive for HCV-RNA and/or anti-HCV antibody confirmation test were defined as having HCV infection. Those with detectable HCV RNA were subsequently tested for HCV genotype. In 2012 and earlier years, the HCV testing algorithm began with HCV antibody screening test and followed by HCV confirmation test (Fig 1B). Specimens that were anti-HCV antibody positive in both screening and confirmation tests were tested by HCV-RNA test. Anti-HCV antibody screening tests were performed by direct solid-phase enzyme immunoassay (1999–2008) or VITROS anti-HCV assay (Ortho-Clinical Diagnostics, Rochester, NY, USA) (2009–2012). Confirmation tests were performed by the Chiron RIBA Processor System (Chiron Corporation, Inc.). HCV RNA was performed by reverse transcriptase-PCR amplification of the 5’ noncoding region. Individuals with a positive anti-HCV antibodies and detectable HCV RNA were classified as having chronic HCV, while those with undetectable HCV RNA were defined as resolved HCV infection (Fig 1B). Beginning in 2013, samples reactive to the antibody screening test were first tested for RNA, followed by HCV confirmation test (Fig 1C). This change was made to align the HCV testing algorithm with a 2013 update to guidance of the U.S. Centers for Disease Control and Prevention (CDC) on testing for HCV infection [18]. Anti-HCV screening test was performed by VITROS Immunodiagnostic Products aHCV and confirmatory test was performed by Fujirebio INNO-LIA HCV Score Assay. Participants who were tested negative for HCV antibody screening were defined as no HCV infection. Those who were tested positive for HCV screening were further tested by HCV RNA test. Individuals with a positive anti-HCV antibody screening tests and detectable HCV RNA was defined as chronic HCV infection, while those without RNA were further tested by confirmation test. A positive result was defined as resolved HCV infection, while an indeterminate or negative result was defined as no history of HCV infection (Fig 1C).

The outcome of interest was kidney disease, which was defined as prevalent eGFR < 60 mL/min/1.73 m^2^ or urinary albumin creatinine ratio (ACR) ≥ 30 mg/g. The eGFR was calculated by CKD Epidemiology Collaboration (CKD-EPI) creatinine equation [19]. The urinary albumin was measured by a solid-phase fluorescent immunoassay. The urinary creatinine was measured by colorimetric method (Jaffe rate method) before 2016 and by enzymatic method after 2017.

Other covariates included histories of diabetes mellitus or hypertension, which were defined as self-reporting diagnosis with the disease or taking medications. Cardiovascular disease (CVD) was defined by self-reported history of congestive heart failure, coronary heart disease, angina, or heart attack; previous stroke was also defined by self-reported history of the diseases. Race/ethnicity was categorized as non-Hispanic Whites, non-Hispanic Blacks, Hispanics, and other race including multi-racial by self-report. Body mass index (BMI) was calculated as body weight in kilograms divided by the square of height in meters. Human immunodeficiency virus (HIV) infection was defined as positive HIV antibody test. Chronic hepatitis B (CHB) was defined as positive hepatitis B surface antigen or positive hepatitis B core antigen with negative hepatitis B surface antibody.

### Statistical analysis

Continuous variables were presented as mean ± standard deviation and were tested by independent sample *t* test. Categorical variables were presented as numbers (percent) and were compared by χ^2^ tests. Tests were two-tailed with a significance level of 0.05. Logistic regression analysis was performed to explore the association between HCV infection and kidney disease. In multivariable logistic regression, we adjusted for age, sex, self-identified race/ethnicity (Black or non-Black), BMI, as well as self-reported diabetes, hypertension, CVD, previous stroke, and HIV infection, all of which were known risk factors of kidney disease. We also performed multiple logistic regression analysis to explore the effect of HCV genotype 1 versus non-genotype 1 on kidney disease risk with adjustment of age, sex, diabetes, hypertension, CVD, and HIV infection; the variable selection in the regression model was based on previously reported factors associated with kidney disease and backward stepwise selection method of the software because of relatively small sample size. Data were presented as odds ratio (OR) and 95% confidence interval (CI). Statistical computation was performed using IBM SPSS V22.

## Results

The average age of the study population was 46.7±17.0 years old and 49.8% of them were males, with race distribution of 42.0% non-Hispanic Whites, 21.2% non-Hispanic Blacks, and 27.5% Hispanics (Table 1). Among the 44,998 participants, the prevalence of resolved and chronic HCV infection was 0.6% (255 individuals) and 1.3% (586 individuals), respectively. Compared with individuals without HCV infection (n = 44,157), those with resolved or chronic HCV infection tended to be older and male predominant, and were more likely to have hypertension, CVD, previous stroke, chronic hepatitis B, and HIV infection. There was no significant difference in prevalence of low eGFR (< 60 mL/min/1.73 m^2^) among the three groups, while the prevalence of albuminuria was higher in those with resolved or chronic HCV infection (Table 1).

**Table 1:** Characteristics of study participants by hepatitis C virus (HCV) infection status.

		Hepatitis C virus (HCV) infection status			
	Total	no HCV	resolved HCV	chronic HCV	*p* value
No. of participants	n = 44998	n = 44157	n = 255	n = 586	
Age (years old)	46.7±17.0	46.6±17.1	51.5±12.7	51.9±10.4	<0.001
Age group					<0.001
≤39	16868 (37.5%)	16741 (37.9%)	53 (20.8%)	74 (12.6%)	
40–59	15317 (34.0%)	14841 (33.6%)	120 (47.1%)	356 (60.8%)	
≥60	12813 (28.5%)	12575 (28.5%)	82 (32.2%)	156 (26.6%)	
Male	22418 (49.8%)	21874 (49.5%)	148 (58.0%)	396 (67.6%)	<0.001
Race					<0.001
Whites	18885 (42.0%)	18535 (42.0%)	122 (47.8%)	228 (38.9%)	
Black	9562 (21.2%)	9276 (21.0%)	53 (20.8%)	233 (39.8%)	
Hispanics	12387 (27.5%)	12223 (27.7%)	61 (23.9%)	103 (17.6%)	
Others	4164 (9.3%)	4123 (9.3%)	19 (7.5%)	22 (3.8%)	
BMI (kg/m^2^)	29.0±6.8	29.0±6.8	29.3±6.8	27.8±6.4	<0.001
SBP (mmHg)	123.5±18.3	123.5±18.2	125.1±17.6	128.0±20.4	<0.001
DBP (mmHg)	71.2±11.7	71.1±11.7	72.6±11.4	75.8±13.3	<0.001
Diabetes	5312 (11.8%)	5199 (11.8%)	39 (15.3%)	74 (12.6%)	>0.05
Hypertension	14354 (31.9%)	13964 (31.7%)	119 (46.7%)	271 (46.5%)	<0.001
CVD	3158 (7.0%)	3065 (6.9%)	35 (13.7%)	58 (9.9%)	<0.001
Previous stroke	1306 (2.9%)	1255 (2.8%)	19 (7.5%)	32 (5.5%)	<0.001
CHB	238 (0.5%)	228 (0.5%)	4 (1.6%)	6 (1.0%)	0.017
HIV infection	156 (0.3%)	138 (0.3%)	7 (2.7%)	11 (1.9%)	<0.001
Creatinine (mg/dL)	0.87±0.29	0.87±0.29	0.88±0.25	0.91±0.30	<0.001
eGFR	97.5±22.8	97.5±22.8	94.3±20.1	95.3±20.4	<0.01
Low eGFR (eGFR < 60)	2546 (5.7%)	2497 (5.7%)	17 (6.7%)	32 (5.5%)	>0.05
Albuminuria	5027 (11.2%)	4874 (11.0%)	50 (19.6%)	103 (17.6%)	<0.001
Albumin (g/dL)	4.27±0.34	4.27±0.34	4.18±0.33	4.06±0.40	<0.001
AST (IU/L)	25.5±19.2	25.0±18.2	27.2±22.6	57.2±45.5	<0.001
ALT (IU/L)	25.7±25.0	25.3±23.5	24.7±21.8	61.3±69.4	<0.001
T-Bilirubin (mg/dL)	0.67±0.31	0.67±0.31	0.61±0.37	0.74±0.38	<0.001
Cholesterol (mg/dL)	194.5±41.9	194.7±41.9	190.7±45.2	178.3±37.9	<0.001
Glucose (mg/dL)	101.2±38.5	101.1±38.3	104.0±40.3	104.9±48.0	<0.05
Uric acid (mg/dL)	5.43±1.43	5.42±1.43	5.51±1.51	5.71±1.44	<0.001
TG (mg/dL)	150.5±134.3	150.8±134.8	158.1±134.7	129.4±87.8	<0.001

BMI: Body mass index; SBP: Systolic blood pressure; DBP: Diastolic blood pressure; CVD: Cardiovascular disease; CHB: Chronic hepatitis B; HIV: Human immunodeficiency virus; eGFR: Estimated glomerular filtration rate (ml/min/1.73 m^2^); AST: Aspartate aminotransferase; ALT: Alanine aminotransferase; T-Bilirubin: Total bilirubin; TG: Triglyceride.

Among the overall population, the crude prevalence of kidney disease was 14.9% (6,706 out of 44,998 individuals). Compared with individuals without HCV infection, those with resolved or chronic HCV infection had higher prevalence of kidney disease: 14.8% (6,528 individuals) vs. 23.5% (60 individuals) vs. 20.1% (118 individuals), *p*<0.001. In addition, when dividing the study population based on the survey cycles into 1999–2002, 2003–2006, 2007–2010, 2011–2014, and 2015–2018, we showed no significant differences in secular trends in kidney disease risk among HCV-infected individuals (S1 Fig). By multivariable logistic regression, we showed that both resolved (OR: 1.40, 95% CI: 1.02–1.93, *p*<0.05) and chronic HCV infection (OR: 1.26, 95% CI: 1.01–1.57, *p*<0.05) were associated with increased risk of kidney disease compared with no HCV infection after adjusting for age, sex, race (Black vs. non-Black), BMI, diabetes, hypertension, CVD, previous stroke, and HIV infection (Table 2).

**Table 2:** Risk factors of kidney disease among total population by multivariable logistic regression.

	Odds ratio	95% Confidence Interval		*p* value
		Lower limit	Upper limit	
Age (every 10-year-old increment)	1.43	1.40	1.46	<0.001
Sex (male vs. female)	0.86	0.82	0.91	<0.001
Race/ethnicity (Black vs. non-Black)	1.11	1.04	1.19	<0.01
BMI	1.01	1.01	1.02	<0.001
Diabetes (yes vs. no)	2.51	2.34	2.69	<0.001
Hypertension (yes vs. no)	1.63	1.53	1.74	<0.001
CVD (yes vs. no)	1.67	1.53	1.82	<0.001
Previous stroke (yes vs. no)	1.59	1.40	1.81	<0.001
HIV infection (yes vs. no)	1.72	1.09	2.72	<0.05
HCV infection status				
No HCV	1	1	1	
Resolved HCV	1.40	1.02	1.93	<0.05
Chronic HCV	1.26	1.01	1.57	<0.05

BMI: Body mass index; CVD: Cardiovascular disease; HIV: Human immunodeficiency virus; HCV: Hepatitis C virus.

**Fig 2:**
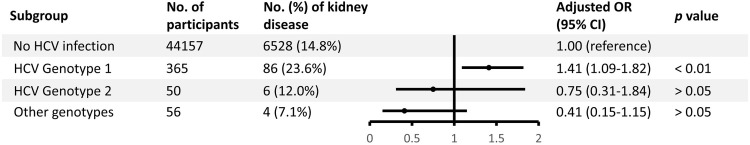
Hepatitis C virus (HCV) genotype 1 correlated to increased kidney disease risk. Compared with no HCV infection, we showed that HCV genotype 1 but not genotype 2 or other genotypes correlated to increased kidney disease risk after adjusted for age, sex, self-identified race/ethnicity (Blacks or non-Blacks), body mass index (BMI), diabetes, hypertension, cardiovascular disease, previous stroke, and human immunodeficiency virus (HIV) infection.

Among individuals with detectable HCV RNA (n = 586), genotype 1 accounted for 62.3% (365 individuals), genotype 2 accounted for 8.5% (50 individuals), other genotypes accounted for 9.6% (56 individuals), with 19.6% (n = 115) missing genotype tests. The crude prevalence of kidney disease among individuals with HCV genotype 1, genotype 2, and other genotypes were 23.6% (86 out of 365 individuals), 12.0% (6 out of 50 individuals), and 7.1% (4 out of 56 individuals), respectively (*p*<0.01). When compared with no HCV infection, our results showed that HCV genotype 1 was significantly associated with kidney disease (adjusted OR: 1.41, 95% CI: 1.09–1.82, *p*<0.01) after adjusting for age, sex, race (Black vs. non-Black), BMI, diabetes, hypertension, CVD, previous stroke, and HIV infection, but genotype 2 and other genotypes did not correlate to increased kidney disease risk (Fig 2).

**Fig 3:**
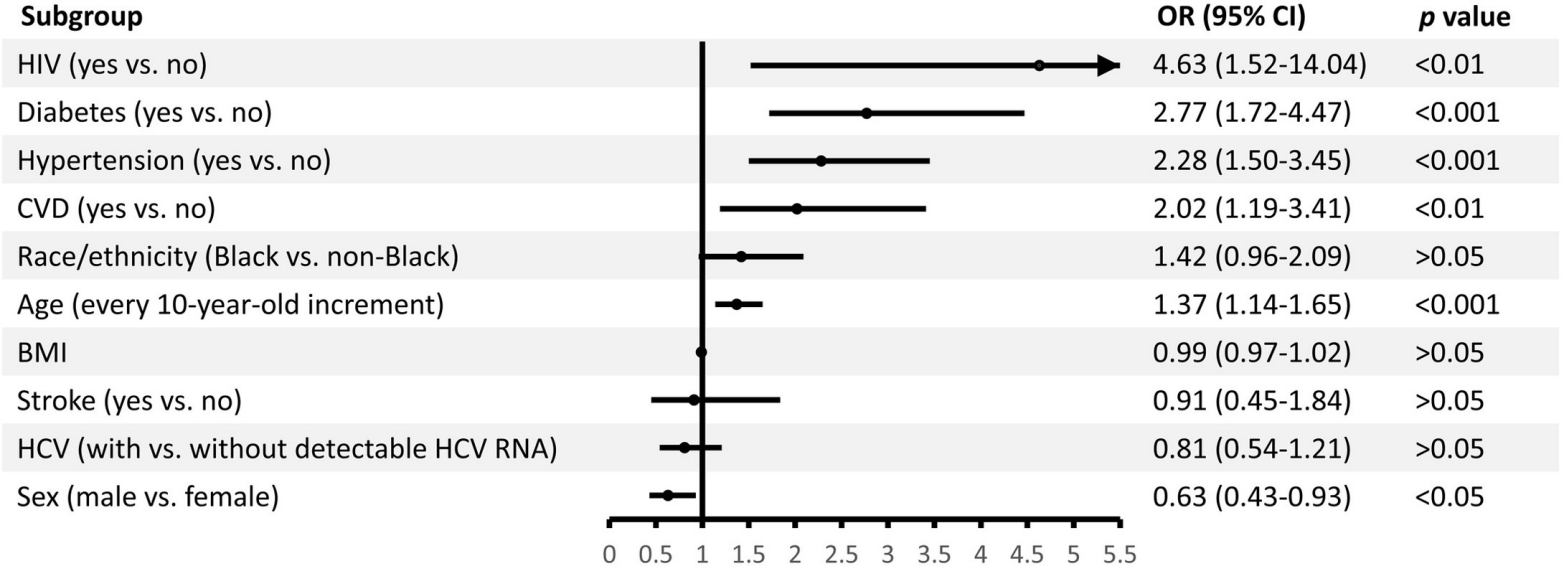
No difference in kidney disease risk between those with and without detectable hepatitis C virus (HCV) ribonucleic acid (RNA). Variables in the multivariable logistic regression model include age, sex, self-identified race/ethnicity (Blacks or non-Blacks), body mass index (BMI), diabetes, hypertension, cardiovascular disease (CVD), previous stroke, HCV infection tatus (detectable or undetectable HCV RNA), and human immunodeficiency virus (HIV) infection.

Among individuals with HCV infection, the independent risk factors of kidney disease included HIV co-infection, diabetes, hypertension, CVD, being female, and older age, while there was no difference in risk of kidney disease between those with and without detectable HCV RNA (Fig 3). When compared HCV genotype 1 with non-genotype 1, we showed that genotype 1 was associated with greater risk of kidney disease (adjusted OR: 2.20, 95% CI: 1.07–4.53, *p*<0.05) after adjusting for age, sex, diabetes, hypertension, CVD, and HIV infection (Table 3).

**Table 3:** Risk factors of kidney disease among individuals with detectable hepatitis C virus ribonucleic acid (RNA) by multivariable logistic regression.

		95% Confidence Interval		
	Odds ratio	Lower limit	Upper limit	*p* value
Age (every 10-year-old increment)	1.67	1.26	2.21	<0.001
Sex (male vs. female)	0.53	0.32	0.89	<0.05
Diabetes (yes vs. no)	2.71	1.46	5.02	<0.01
Hypertension (yes vs. no)	2.33	1.33	4.07	<0.01
CVD (yes vs. no)	2.13	1.09	4.17	<0.05
HIV infection (yes vs. no)	8.34	1.40	49.59	<0.05
HCV genotype (genotype 1 vs. non-genotype 1)	2.20	1.07	4.53	<0.05

CVD: Cardiovascular disease; HIV: Human immunodeficiency virus; HCV: Hepatitis C virus.

## Discussion

In this population-based study, we showed that individuals with resolved and chronic HCV infection were 40% and 26% more likely to have kidney disease, respectively, when compared with non-HCV infected individuals. In addition, our results showed that genotype 1 HCV infection was associated with 41% higher risk of kidney disease when compared with non-HCV infected individuals, while genotype 2 and other genotypes did not have a significant effect. Furthermore, among individuals with HCV infection, we observed a 2-fold greater risk of kidney disease in individuals with genotype 1 HCV infection compared with non-genotype 1 HCV infection.

In our study, we observed that the risk of kidney disease was similar for those with and without detectable HCV RNA. HCV may lead to kidney injury through formation of immune complexes or direct cytopathic effect. Deposition of immune complexes and inflammatory cascade were responsible for development of glomerulonephritis and ensuing kidney disease [1]. Therefore, we rationally speculated that, despite HCV infection resolved spontaneously or by medical treatment, obsolete injury to glomeruli still raises the risk of kidney disease comparable to chronic HCV infection. Although several studies had demonstrated that sustained virologic response from treatment of HCV with direct-acting antiviral agents (DAAs) or interferon-based therapies was associated with reduced risk of progressive kidney disease [10,11], our findings suggested that individuals with resolved HCV infection still require medical attention and prompt assessment of kidney disease risk.

DAAs have been used in daily clinical practice for nearly a decade in the world. Several studies showed that use of sofosbuvir-based DAAs was associated with eGFR decline during the on-treatment period [20–22]. However, when follow up for additional 24 weeks, patients receiving sofosbuvir-based DAAs showed improvement in eGFR during off-treatment period, although still worse compared to sofosbuvir-free regimen [20]. As our study showed that there is no significant secular trend in kidney disease risk among HCV-infected individuals, it is unclear whether the widely used sofosbuvir-based DAAs attenuates the beneficial effect of HCV treatment on kidney function. Longitudinal research with longer follow-up period will be needed to elucidate the role of sofosbuvir-based and sofosbuvir-free DAAs on the long-term evolution of kidney function.

The impact of various HCV genotypes on kidney disease is rarely examined. Early studies showed that the risk of progressive kidney disease did not vary between genotype 1 and other genotypes among human immunodeficiency virus (HIV)-infected adults with HCV co-infection [13,14]. In contrast, our study showed that HCV genotype 1 correlated to risk of kidney disease, but not genotype 2 or other genotypes. A cohort study also demonstrated the association between genotype 1 HCV infection and progressive kidney disease but not genotype 2 [16], which was consistent with our findings. However, a large-scale community-based study indicated that both genotypes 1 and genotype 2 HCV were associated with increased risk of CKD, with impact of genotype 2 being stronger than genotype 1 [15]. Future research will be needed to elucidate the impact of various HCV genotypes on kidney disease risk.

Although several previous research have demonstrated an association between HCV infection and kidney disease risk, studies with inconsistent results have also been observed [7–9,23]. Additionally, while several studies have shown that HCV treatment is associated with a reduced risk of progressive kidney disease [10,11], some studies have not yielded a positive finding in eGFR improvement with HCV treatment [24,25]. As our findings showed that different HCV genotypes have different effects on kidney disease, one question is whether the conflicting results in previous studies are due to the differences in HCV genotypes. However, the effect of HCV genotype on incident CKD or end-stage kidney disease, or evolution in eGFR following antiviral treatment have been demonstrated, which did not yield difference among patients infected with different HCV genotypes [26,27].

Our study examined the association of HCV genotypes on kidney disease, which had rarely been investigated in previous studies. Our results showed a stronger effect of genotype 1 than genotype 2 or other genotype, providing insight into this insufficiently researched area. However, there are several study limitations that should be considered in the interpretation of our study. First, HCV viral load has been shown to be a determinant of kidney damage. While high titers of HCV RNA were significantly associated with progressive kidney disease, the effect attenuated in individuals with low viral load [15,16]. In our study, we did not quantify HCV RNA level in individuals with chronic HCV infection, and hence it is unclear whether the null association between HCV genotype 2 or other genotypes and kidney disease is confounded by low viral load. Second, duration of HCV infection was unknown, and it was unclear whether individuals with resolved HCV infection were due to spontaneous viral clearance or treatment-induced viral clearance, which represented significant clinical implications and had different risks of HCV exposure. Patients with spontaneous viral clearance may have very short-term HCV exposure, therefore, the risk of HCV on kidney damage may be trivial. In contrast, patients with treatment-induced viral clearance may have years of HCV exposure, which posed a similar risk of renal decline to that in patients with chronic HCV infection. Third, this is a cross-sectional study, and hence the temporality cannot be established. However, HCV infection is usually acquired early in life, and people with HCV infection are usually asymptomatic. Therefore, we speculated that the HCV-infected participants in our study population had lived with HCV for several years before developing kidney disease. Fourth, because this is an observational study, the causal relationship cannot be confirmed. For example, the secular changes in renal function or causative events of acute kidney injury are lacking in our study. Fifth, because of low prevalence of HCV infection in the U.S., we merged 1999–2000 through the 2017–2018 cycle of the NHANES data. The prolonged time frame of enrollment may result in repeated measurements of participants. However, because of the low prevalence of HCV infection, the probability of repeated measurement of HCV-infected participants is lower than non-HCV infected participants. This may lead to underestimation of the effect of HCV infection as our study showed that HCV infection is associated with higher risk of kidney disease. Sixth, because clinical data such as diabetes, hypertension, and CVD was ascertained from participant report, recall bias possibly impacts the accuracy of our analysis. Finally, although we have adjusted for several potential confounders in our regression analyses, residual confounding effect may have existed and bias our study results.

In conclusion, our study demonstrated that both resolved and chronic HCV infection were associated with increased risk of kidney disease, suggesting that individuals without detectable HCV RNA still require medical attention and assessment of kidney disease risk. In addition, among individuals with chronic HCV infection, those with HCV genotype 1 but not genotype 2 or other genotype were more likely to have kidney disease. Future studies are warranted to determine the impact of various genotypes on kidney disease risk.

## Supporting information

S1 Fig No significant differences in secular trend of kidney disease prevalence among individuals with resolved and chronic hepatitis C virus (HCV) infection, respectively. (PDF)
