# Supplementary material for: Association of hepatitis C virus infection status and genotype with kidney disease risk: A population-based cross-sectional study
Source: PLoS One. 2022 Jul 8;17(7):e0271197. doi: 10.1371/journal.pone.0271197 (PMC9269772; doi:10.1371/journal.pone.0271197)
Supplement: S1 Fig — (PDF) [file pone.0271197.s001.pdf]

Association of hepatitis C virus infection status and genotype with kidney disease risk: a population-based cross-sectional study

Yi-Chia Chen M.D. [1], Hung-Wei Wang M.D. [2], Yun-Ting Huang M.D. [3], Ming-Yan Jiang M.D., MSc [3,4]

[1] Department of Internal Medicine, Chi Mei Medical Center, Tainan, Taiwan

[2] Renal division, Department of Internal Medicine, Chi Mei Hospital Chiali, Tainan, Taiwan

[3] Renal division, Department of Internal Medicine, Chi Mei Medical Center, Tainan, Taiwan

[4] Department of Pharmacy, Chia Nan University of Pharmacy & Science, Tainan, Taiwan

Supplementary Figure S1. No significant differences in secular trend of kidney disease prevalence among individuals with resolved and chronic hepatitis C virus (HCV) infection, respectively.

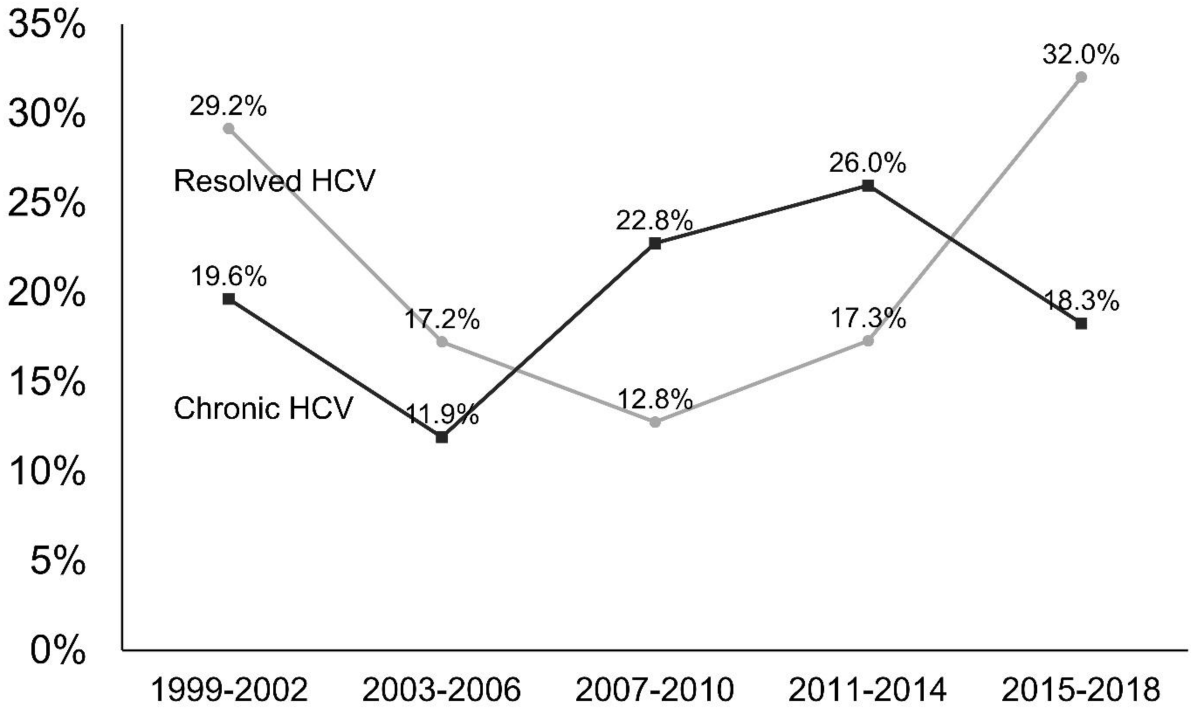

	1999-2002	2003-2006	2007-2010	2011-2014	2015-2018	*p* value
Resolved HCV	7/24	5/29	6/47	9/52	33/103	> 0.05
Chronic HCV	22/112	13/109	33/145	33/127	17/93	> 0.05
